# Bezlotoxumab in Patients with a Primary *Clostridioides difficile* Infection: A Literature Review

**DOI:** 10.3390/antibiotics11111495

**Published:** 2022-10-28

**Authors:** Guido Granata, Francesco Schiavone, Giuseppe Pipitone

**Affiliations:** 1 Clinical and Research Department for Infectious Diseases, National Institute for Infectious Diseases L. Spallanzani, IRCCS, 00149 Rome, Italy; 2 Divers and Raiders Group Command “Teseo Tesei” COMSUBIN, Medical Service, Italian Navy, 19025 Portovenere, Italy; 3 Infectious Disease Unit, ARNAS Civico-Di Cristina, Piazza Leotta, 5, 90100 Palermo, Italy

**Keywords:** primary CDI, *Clostridioides difficile*, TcdB, toxin, bezlotoxumab, recurrence of CDI, CDI recurrence prevention, cost-effectiveness, prevention, health care cost

## Abstract

Background: Nowadays, one of the main issues in the management of *Clostridioides difficile* infection (CDI) is the high rate of recurrences (rCDI), causing increased mortality and higher health care costs. Objectives: To assess the available evidence on the use of bezlotoxumab for the prevention of rCDI during a first CDI episode. Methods: Published articles on bezlotoxumab during a primary CDI episode were identified through computerized literature searches with the search terms [(bezlotoxumab) AND (CDI) OR (*Clostridioides difficile* infection)] using PubMed and by reviewing the references of retrieved articles. PubMed was searched until 31 August 2022. Results: Eighty-eight studies were identified as published from December 2014 to June 2022. Five studies were included in this study, one was a phase III clinical trial and four were sub-analyses or extensions of the previous phase III clinical trial. In the phase III clinical trial, the subgroup analysis on the included primary CDI patients showed that 13.5% of patients receiving bezlotoxumab had an rCDI, whilst 20.9% of patients in the placebo group had an rCDI at the twelve weeks follow-up (absolute difference: −7.4). Conclusions: Bezlotoxumab administration during the standard of care antibiotic therapy is effective and safe in reducing the rate of rCDI. Despite its high cost, evidence suggests considering bezlotoxumab in patients with a primary CDI episode. Further studies are needed to assess the benefit in specific subgroups of primary CDI patients and to define the risk factors to guide bezlotoxumab use.

## 1. Introduction

The Gram-positive anaerobic bacterium *Clostridioides difficile* is among the main pathogens responsible for nosocomial diarrhea, causing significant morbidity, mortality, prolonged hospital stay and high healthcare costs worldwide [1,2,3,4,5,6,7]. A relevant issue during CDI is its high rate of recurrences (rCDI). Clinical studies show wide-ranging rCDI rates after the primary CDI, between 10% and 30% [8,9,10,11]. rCDI is associated with a higher risk of death and higher hospitalization costs [1,2,3,4,5,6,7]. Currently, the main approaches to treat a primary CDI are the oral anti-*Clostridioides difficile* antibiotics vancomycin or fidaxomicin, while oral metronidazole should be used only when vancomycin and fidaxomicin are not available or feasible. For rCDI, additional non-antimicrobial approaches may be considered, i.e., fecal microbiota transplant or bezlotoxumab. Bezlotoxumab is a monoclonal antibody directed against *Clostridioides difficile* toxin B, effective in reducing the rate of further rCDI. One of the main drawbacks of bezlotoxumab is its high cost.

In the recently released updates of guidelines/guidance documents from the Infectious Diseases Society of America/Society for Healthcare Epidemiology of America (IDSA/SHEA) and from the European Society of Clinical Microbiology and Infectious Diseases (ESCMID), there have been changes in the recommendations pertaining to the use of bezlotoxumab [12,13]. The IDSA/SHEA guidelines recommend that in settings where logistics is not an issue, patients with a primary CDI episode and other risk factors for rCDI and severe CDI on presentation may particularly benefit from receiving bezlotoxumab. The ESCMID guidelines recommend considering bezlotoxumab in addition to standard-of-care antibiotics for the treatment of a second or further rCDI.

We performed a literature review with the main aim of summarizing available evidence on the use of bezlotoxumab during a first CDI episode to prevent rCDI.

## 2. Materials and Methods

### Search strategy and Article Identification

Published articles (from June 2017 to November 2020) assessing the efficacy and safety of bezlotoxumab for the prevention of rCDI after a first CDI episode were identified through computerized literature searches using PubMed until 31 August 2022. A combination of the following search terms was used: [(Bezlotoxumab) AND (*Clostridioides difficile*) OR (CDI)]. English language restriction was applied. 

Randomized clinical trials and original research articles reporting original data on the use of bezlotoxumab during a first CDI episode were included in this study. Studies published only in abstract form, correction articles, reviews, case reports, editorials, guidance articles or guidelines and clinical trial protocols were not included.

Quantitative and qualitative information from the included studies was summarized by means of textual descriptions.

## 3. Results

### Studies Description

**Figure 1:**
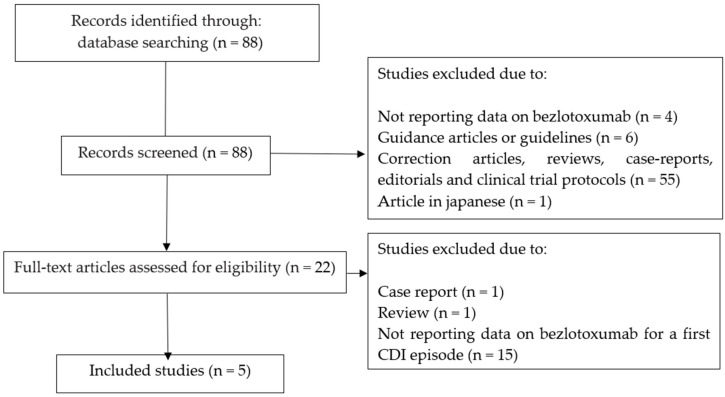
Flowchart depicting the selection process of studies included in this study.

Figure 1 shows the selection process of the included studies. Through a PubMed search with the search terms “bezlotoxumab” and “*Clostridioides difficile*” or “CDI”, we identified 88 studies published from December 2014 to June 2022. Of the 22 full-text articles assessed for eligibility, fifteen studies were excluded because they did not report data on bezlotoxumab for a first CDI episode, one study was excluded because it was a “review article” and one study was excluded because it was a “correction article”. The remaining five studies were included in this study (Figure 1) [14,15,16,17,18]. Of the five studies, one was a phase III clinical trial study on the efficacy and safety of bezlotoxumab for the prevention of rCDI, and four studies were sub-analysis or extensions of the previous phase III clinical trial. A summary description of the five included studies is reported in Table 1.

The large randomized, placebo-controlled, phase III trials on bezlotoxumab MODIFY I and MODIFY II showed a substantially lower rate of rCDI than placebo with a comparable safety profile [14]. The primary endpoint of the MODIFY II trial was the proportion of participants with rCDI through 12 weeks of follow-up. The subgroup analysis provided the rCDI rate of the primary CDI patients included in this trial (namely, no CDI episodes in the past six months). Furthermore, 75 out of 556 (13.5%) primary CDI patients receiving bezlotoxumab plus standard of care treatment had an rCDI, whilst 114/545 (20.9%) primary CDI patients in the placebo group had rCDI at the twelve weeks follow-up (absolute difference: −7.4) [14].

A study extending the MODIFY II trial follow-up to twelve months was performed to assess the long-term rates of rCDI and *Clostridioides difficile* colonization following bezlotoxumab infusion. At the end of the twelve-month follow-up of this study, no participants who achieved sustained clinical cure following bezlotoxumab infusion experienced rCDI, whilst only one patient in the placebo group experienced rCDI [15].

A post-hoc analysis of the MODIFY I and II trials was performed to assess bezlotoxumab efficacy in participants with characteristics associated with increased risk for rCDI [16]. In this study, patients enrolled in the MODIFY trials were grouped according to their risk factors for rCDI, including age ≥ 65 years, history of CDI, compromised immunity, severe CDI, and ribotype 027/078/244. Data showed that 424 primary CDI patients were treated with bezlotoxumab and 400 primary CDI patients received placebo. Moreover, 69/424 (16.3%) versus 106/400 (26.5%) had rCDI in 12 weeks, with an absolute difference of −10.1%, significantly favoring bezlotoxumab [16].

A subgroup analysis was performed on the data from the Japanese patients included in the MODIFY trials. In comparison to the general population included in the MODIFY trials, Japanese patients were older (Japanese older than 65 years: 91% versus overall MODIFY patients: 53%) and the proportion of Japanese patients with severe CDI was higher (Japanese: 24%, overall MODIFY patients: 16%). In addition, the proportion of subjects with a prior history of CDI was lower (Japanese: 19/93, 20%, overall MODIFY patients: 28%). Among the 95 Japanese patients, the observed rCDI rate was 46% in the placebo arm versus 21% in the bezlotoxumab arm (*p*: 0.0197) [17].

A study was performed to assess the cost-effectiveness of bezlotoxumab, compared with standard of care alone, in subgroups of CDI patients included in the MODIFY trials [18]. This study adopted a computer-based Markov health state transition model to track the natural history of patients infected with CDI. The simulation followed the cohort over a lifetime horizon, and costs and utilities for the various health states were used to estimate incremental cost-effectiveness ratios. Regarding the subgroup of CDI patients with no previous CDI episodes in the past six months, the cost-effectiveness model showed that, compared with placebo, bezlotoxumab could reduce rCDI by 10.1% (26.6% versus 16.5%), and the 180-day mortality by 1.1%. In this model, bezlotoxumab was also cost-effective in preventing rCDI recurrences [18].

## 4. Discussion

Nowadays, CDI and rCDI remain associated with a reduction in patient quality of life and with increased healthcare costs. Bezlotoxumab is a promising option to reduce the burden of rCDI. The randomized, placebo-controlled phase III trial MODIFY II showed a substantially lower rate of rCDI in patients treated with bezlotoxumab [14]. Nonetheless, experiences outside randomized controlled trials remain scant. The MODIFY trial has the limitation that the target population was a selected sample of participants with a low prevalence of multiple risk factors for recurrence. 

Currently, the most recent international guidelines differ in the recommendations regarding the use of bezlotoxumab for the first episode of CDI and rate the certainty of the evidence as only moderate [12,13]. The IDSA/SHEA guidelines recommend that in settings where logistics are not an issue, patients with a primary CDI episode and other risk factors for rCDI or severe CDI may receive bezlotoxumab despite its high cost [12]. Differently, the ESCMID guidelines recommend considering bezlotoxumab in addition to standard-of-care antibiotics only for the treatment of a second or further rCDI.

Importantly, it has to be kept in mind that in patients with a history of congestive heart failure, bezlotoxumab should be reserved for use when the benefits outweigh the risk [12,13].

Nevertheless, it is promising that in the MODIFY II trial, the estimated number needed to treat to prevent one episode of rCDI after a primary CDI episode with bezlotoxumab was 10 [14]. Interestingly, available data suggest that the efficacy of bezlotoxumab is due to rCDI prevention rather than a delay in rCDI onset after antibody concentrations were diminished [15]. Moreover, in post-hoc analyses of the MODIFY trials, bezlotoxumab reduced the rate of rCDI even in the group of patients with a primary CDI and no risk factors for rCDI [16,17].

Despite the growing data evidence supporting the use of bezlotoxumab to prevent rCDI, its use in many European countries is still limited and restricted to participants who experienced previous CDI episodes. This might be mainly explained by the direct drug cost of bezlotoxumab. However, studies adopting cost-effectiveness models show that for preventing rCDI recurrences, bezlotoxumab may be cost-effective [18].

It is likely that the overall future scenario may change from “administer bezlotoxumab only in high-risk patients, because of the high cost of this compound” to “if feasible, consider bezlotoxumab even for a primary CDI episode, in view of the global benefits for the patient and the cost-effectiveness provided by the reduction of the rate of the expensive rCDI episodes”. In our opinion, future studies are needed to clarify some remaining unanswered questions: First, the efficacy of fidaxomicin plus bezlotoxumab in preventing rCDI in comparison to vancomycin plus bezlotoxumab. Second, the use of bezlotoxumab in specific, high-risk subgroups of patients experiencing a primary CDI, i.e., hematologic patients, hematopoietic cell transplantation patients, patients receiving immunosuppression after solid organ transplantation, patients with impairment of humoral immunity. Third, bezlotoxumab use in patients with severe CDI.

## 5. Conclusions

Data coming from the first available research studies show that bezlotoxumab administration during the standard of care antibiotic therapy is effective and safe in reducing the rate of further rCDI. Despite its high cost, this evidence suggests considering bezlotoxumab not only among patients with multiple CDI episodes, but also in patients with a primary CDI episode.

Further studies are needed to assess the exact benefit associated with bezlotoxumab in specific subgroups of primary CDI patients and to define the risk factors to guide bezlotoxumab use.

## Figures and Tables

**Table 1:** Summary description of the 5 studies providing data on the use of bezlotoxumab for the prevention of rCDI after a first CDI episode.

Author	Country	Study Design	Study Aim	Methods	Study Results
Wilcox M et al., 2017 [14]	30 different Countries	Placebo-controlled, double-blind, single-infusion, phase III clinical trial	To evaluate the efficacy and safety of bezlotoxumab (alone and in combination with actoxumab) for the prevention of rCDI	2655 adult patients with primary or rCDI were randomized 1:1:1 to receive 60 min intravenous infusion of bezlotoxumab (10 mg/kg), actoxumab plus bezlotoxumab (10 mg/kg each) or placebo during the standard of care antibiotic therapy Primary endpoint was the proportion of participants with rCDI during 12 weeks of follow-up in the modified intention-to-treat population	Rate of rCDI was lower with bezlotoxumab than with placebo (MODIFY II: 16% vs. 26%, *p* < 0.001) The subgroup analysis providing the rCDI rate among primary CDI patients showed that 75/556 (13.5%) patients receiving bezlotoxumab plus standard-of-care treatment had an rCDI, whilst 114/545 (20.9%) patients in the placebo group had rCDI at the twelve weeks follow-up (absolute difference: −7.4)
Goldstein EJC et al., 2020 [15]	30 different Countries	Extension of MODIFY II clinical trial	To assess the long-term rates of rCDI and *Clostridioides difficile* colonization following bezlotoxumab infusion	The study included 293 participants of MODIFY II who provided stool samples at 6, 9 and 12 months. *Clostridioides difficile* colonization at months 6, 9 and 12 was assessed based on whether a toxigenic *Clostridioides difficile* strain was isolated in samples	At 12 months, the incidence of rCDI in the bezlotoxumab and placebo groups was 18.8% and 51.5% respectively. *Clostridioides difficile* colonization rates were 16–24% in the bezlotoxumab group and 19–32% in the placebo groups
Gerding DN et al., 2018 [16]	30 different Countries	Sub-analysis of the MODIFY I-II clinical trials	To evaluate the efficacy of bezlotoxumab in reducing rCDI among patients with characteristics associated with increased risk factors for rCDI	Patients treated with bezlotoxumab vs. placebo were stratified by risk factors The efficacy was evaluated as: a) achieving initial clinical cure rate, b) reducing the rate of rCDI and c) reducing the rate of FMT	Bezlotoxumab did not affect initial clinical cure rate; bezlotoxumab reduced the rate of rCDI compared to the low-risk group Among primary CDI patients, 69/424 (16.3%) patients treated with bezlotoxumab versus 106/400 (26.5%) controls had rCDI at 12 weeks (absolute difference: −10.1%)
Mikamo H et al., 2018 [17]	Japan	Sub-analysis of the MODIFY I-II clinical trial	To evaluate the efficacy of bezlotoxumab and actoxumab in reducing rCDI rate at week 12	95 Japanese patients were randomized to bezlotoxumab, actoxumab plus bezlotoxumab or placebo in a 1:1:1 ratio Vancomycin, metronidazole and fidaxomicin were administered as standard-of-care antibiotic treatment	The rCDI rate was lower in the bezlotoxumab group (21%) compared to placebo (46%), *p*: 0.0197
Prabhu VS et al., 2018 [18]	30 different Countries	Sub-analysis of MODIFY I-II clinical trial	To assess the cost-effectiveness of bezlotoxumab in subgroups of patients at risk of rCDI	The computer simulation followed the cohort over a lifetime, and healthcare services costs were compared to estimate the incremental cost-effectiveness ratios	In the subgroup of patients with no previous CDI episodes in the past six months, the cost-effectiveness model showed that, compared with placebo, bezlotoxumab could reduce rCDI by 10.1% (26.6% versus 16.5%), and the 180-day mortality by 1.1% Bezlotoxumab was associated with a gain in quality-adjusted life-years and was cost-effective

rCDI: recurrence of CDI; FMT: fecal microbiota transplant.

## Data Availability

The data presented in this study are openly available in the MEDLINE database.
